# Comparative mapping of sequence-based and structure-based protein domains

**DOI:** 10.1186/1471-2105-6-77

**Published:** 2005-03-25

**Authors:** Ya Zhang, John-Marc Chandonia, Chris Ding, Stephen R Holbrook

**Affiliations:** 1 Physical Biosciences Division, Lawrence Berkeley National Laboratory, Berkeley, CA 94720, USA; 2 Computational Research Division, Lawrence Berkeley National Laboratory, Berkeley, CA 94720, USA; 3 School of Information Sciences and Technology, The Pennsylvania State University, University Park, PA 16802, USA

## Abstract

**Background:**

Protein domains have long been an ill-defined concept in biology. They are generally described as autonomous folding units with evolutionary and functional independence. Both structure-based and sequence-based domain definitions have been widely used. But whether these types of models alone can capture all essential features of domains is still an open question.

**Methods:**

Here we provide insight on domain definitions through comparative mapping of two domain classification databases, one sequence-based (Pfam) and the other structure-based (SCOP). A mapping score is defined to indicate the significance of the mapping, and the properties of the mapping matrices are studied.

**Results:**

The mapping results show a general agreement between the two databases, as well as many interesting areas of disagreement. In the cases of disagreement, the functional and evolutionary characteristics of the domains are examined to determine which domain definition is biologically more informative.

## Background

The concept of protein *domains *has gained increasing interest from the biology research community because of its importance in protein classification [1], protein function assignment [2], and protein engineering [3]. Protein domains are generally considered as protein fragments of common structures which may independently fold [4] or have their own functions [5]. They have also been treated as evolutionary units [6]. Protein domains function as the building blocks of proteins and are often recombined to form different proteins [5], leading to high redundancy in protein structures. Currently, a few thousand protein domains have been identified, a total much smaller than the number of proteins. Classifying proteins based on their constituent domains is therefore one of the most effective and efficient approaches to organize protein data both by structures and by evolutionary relationships. However, such a classification requires the identification of domain composition for proteins, which is by no means an easy task. The challenge lies in the ambiguity of domain definitions, as well as the lack of useful structural information about most proteins.

Two types of approaches have been widely used to assign domains: one based on the three-dimensional (3D) structures of proteins and the other based on protein sequences. Structure-based approaches define domains primarily according to the compactness and conservation of protein structural regions, generally described as globular modules. The domain annotation is best achieved through an expert's visual inspection of protein three-dimensional structures. Currently, the Protein Data Bank (PDB) [7], the primary protein structural database, contains 26,610 protein structures. A number of structure-based domain classification databases such as SCOP (Structural Classification of Proteins) [1], FSSP (Families of Structurally Similar Proteins) [8], and CATH (Class Architecture Topology Homology) [9] are constructed using the available protein structures so that proteins can be easily analyzed for the presence of domains. Among them, the SCOP database is manually curated and considered the most reliable domain classification. However, this classification covers only about 2–3% of sequenced proteins. At this time, the Swiss-Prot+TrEMBL [10] sequence databases together contain over 1.5 millon entries. The gap between the number of sequenced proteins and that of proteins with experimentally determined 3D structures is still increasing, which has greatly constrained the development of structure-based protein classification databases. Although 58% of sequences can be modeled using comparative modeling [11], the accuracy of such comparative models decreases sharply below the 30% sequence identity cutoff. An alternative classification schema assigns domains to proteins by only sequence information. Sequence-based domain databases constructed with this classification schema include Pfam [12], ProDom [13] and InterPro [14]. These databases define domains based on sequence similarity and implied evolutionary relationships. In this manuscript we focus on the Pfam database in which domain boundaries are manually assigned by experts.

Since domains are structurally and evolutionarily independent units, we may ask whether either a structure-based or sequence-based classification alone is sufficient and how well they agree. A previous study compared three structure-based classifications: SCOP, CATH and FSSP [15], and concluded that the majority of their classifications agreed. Two sequence-based domain databases were also compared [16] and discrepancies between the two databases were attributed to their different philosophies. In this paper, we strive to improve domain definitions through examining the correspondence between sequence-based domains and structure-based domains, using the domain definitions in SCOP as the representative for structure domains and those of Pfam as the representative for sequence domains. Elofsson and Sonnhammer [17] compared the Pfam and SCOP databases in 1999. According to their comparison, 70% of the SCOP domain families and 57% of the Pfam families have counterparts in the other databases. However, since then, both databases have greatly increased in size and various revisions and updates have been made. For example, the domain representation in Pfam was revised to model discontinuous domains [12]. Therefore, it is now timely and important to revisit this topic and compare the two types of domains under the new setting. Furthermore, the aim of this comparison is to some extent different from what Elofsson and Sonnhammer had. Other than examining the extent that the two databases overlap, we focus more on their differences. When inconsistencies in domain definitions occurs, we propose to determine which domain definition is biologically more meaningful by inspecting the evolution of those domains.

We directly map SCOP domains to Pfam domains based on their corresponding locations in their member sequences. The approach assigns a mapping score to the pair of domains under comparison to quantitatively represent the quality of the match.

The mapping reveals a moderate agreement among Pfam families and SCOP domain families. Five types of relationships between the two classifications are clearly indicated in the mapping results and we therefore put them into five categories. Statistical analysis and individual instances are provided for each category of mapping. In the case of disagreement in domain classification, information from past literature, such as known domain functions, is used as external validation. We also propose to examine the evolutionary history of each individual domain when disagreement occurs.

### An overview of SCOP and Pfam

The SCOP [1] database is manually curated by experts. It orders all proteins with known structures, according to their evolutionary and structural relationships. The database adopts a hierarchical organization: domains are grouped into families, then superfamilies, folds and classes in the highest level of the hierarchy.

Pfam [12] contains hidden Markov model based profiles (HMM-profiles) of many common protein domains based on multiple sequence alignments. While the construction of the HMM-profiles is semi-automatic, expert knowledge contributes in the grouping of proteins, the aligning of protein sequences, and the quality control of the HMM-profiles. Although Pfam is subclassified by 'type' in 2002 as 'family', 'domain', 'repeat' and 'motif', its organization is generally considered to be flat. We hence do not differentiate the subtypes in this comparison.

The Pfam database contains two parts: one is the curated section called Pfam-A and the other is an automatically generated supplement called Pfam-B which represents small families taken from the PRODOM database that do not overlap with Pfam-A. In this study, only Pfam-A families are mapped to SCOP domain families.

## Methods

### Materials

All PDB protein sequences, based on PDB SEQRES records, with less than 95% identity to each other were downloaded from the ASTRAL Compendium [18,19]. This data set contains 8259 protein chains. Pfam 14.0 was downloaded from . Only Pfam-A families were used for the comparison. This version contains 7459 Pfam-A families and corresponding HMM-profiles. The HMMER package, version 2.3.2, was used to compare PDB protein sequences to Pfam-A HMM-profiles. The Pfam 'trusted cutoff' was used to determine whether a Pfam domain matches a PDB chain. The SCOP domain definitions were from the SCOP parsable files version 1.65. Because the SCOP parsable files are based on the PDB ATOM records, the ATOM records were mapped to PDB SEQRES records using the RAF mapping provided by ASTRAL before the comparison.

**Figure 1:**
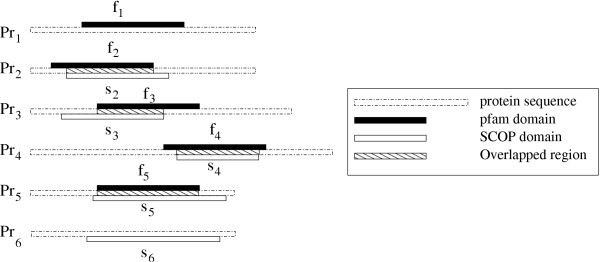
Mapping between Pfam families and SCOP domain families. An instance of a SCOP domain (*s*_*i*_, *i *= 1, ..., 5) on its member sequence is represented by a white rectangle while that of a Pfam domain (*f*_*j*_, *j *= 2, ..., 6) is represented by a black rectangle. Striped rectangles represent their overlap. Location information is used to map a Pfam family and a SCOP domain family. Each Pfam-A family and each SCOP domain family is treated as a set of member protein sequences. The mapping process finds overlapped regions of the two types of domains on their shared member protein sequences. The overlapped regions represent where the two types of domain definitions agree.

We propose to map the Pfam-A families to SCOP domain families based on their locations in member sequences. Each Pfam-A family or SCOP domain family is treated as a set of member protein sequences. A mapping between a Pfam family and a SCOP domain family is defined as follows: (1) they have at least one member protein sequence in common; (2) their locations in the common protein sequences overlap; and (3) their mapping score is larger than the pre-set threshold *m*. For each PDB protein sequence, a comparison was then made for the overlaps and differences in the SCOP domain families and the Pfam families. The process of mapping is illustrated with Figure 1.

### Mapping matrix

**Figure 2:**
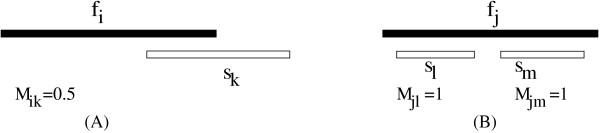
Two cases of domain mapping. An instance of a SCOP domain (s_*_, * = *l*, *m*, *n*) on its member sequence is represented by a white rectangle while that of a Pfam domain (f_*_, * = *i*, *j*) is represented by a black rectangle. (A) A Pfam domain and a SCOP domain overlap at a very small portion of their shared member sequence. This case is considered a partial agreement between the two types of domain definitions, and the mapping score is assigned as 0.5. (B) A Pfam domain overlaps with two SCOP domains over the full lengths of the two SCOP domains, respectively. In this case, we consider the Pfam domain maps to both SCOP domains. Therefore, a score of 1 is assigned to each mapping.

Ideally, if a SCOP domain family and a Pfam family are defined at the same location over the same set of protein chains, then they map exactly to each other. However, in most cases, the mapping is not exact, i.e. they only partially overlap at individual member protein sequences or their member sequences are not all the same. In order to measure the extent of overlap, a mapping score is assigned to each pair of SCOP domain families and Pfam families. Intuitively, if the SCOP domain family and the Pfam family have more members in common and their corresponding protein sequence segments overlap more, then they are more likely to be mapped to each other. However, this mapping criteria favors those domains whose frequencies are high. Since we use only PDB protein chains in the comparative mapping, this data set may be biased towards those proteins of interests to biologists or whose structures are easier to resolve. For both domain models, we observe a power law distribution of domain frequency, where a few domains occurs in a large number of protein sequences and many domains occur in very few protein sequences. To account for the frequencies of domains, the mapping score is normalized by the average frequency of the two domains under comparison. Let *s*_*i *_denotes the *i*-th protein domain in SCOP and *f*_*j *_the *j*-th protein domain in Pfam. The mapping score *M*(*s*_*i*_, *f*_*j*_) is defined as



where *P *represents the set of PDB protein chains with both domain *s*_*i *_and domain *f*_*j*_; *p*_*k *_is the *k*th protein chain in the set; *overlap*(, ) is the length of the overlapped segment on *p*_*k*_; and *length*() is the length of *s*_*i *_on *p*_*k*_. *freq*(*s*_*i*_) and *freq*(*f*_*j*_) represent the frequencies of the *i*th SCOP domain and *j*th Pfam family, respectively. The factor  is to counteract the influence of frequency differences between protein domains. Here *min*(*length*(), *length*()) is used as the denominator because we want to distinguish the cases where two domains overlap in a small part of their coverage and where one domain is completely covered by the other domain, as shown in Figure 2.

### Properties of the mapping matrix

The mapping scores for all SCOP and Pfam domain pairs form a matrix *M*. The matrix representation of the mapping has some nice properties. First consider mapping the SCOP domain *s*_*i *_to all possible Pfam domains. We look at the *i*-th row of *M*. The number of nonzeros, , in the row indicates how many Pfam domains that the SCOP domain *s*_*i *_could possibly map to. Among the possible mapping, the most likely Pfam domain  that the SCOP domain *s*_*i *_will map to is



Note that the number of nonzeros, , could be large, which implies that *s*_*i *_maps to many Pfam domains. However, sometimes, two domains overlap very insignificantly, say only a few amino acid residues. To eliminate the insignificant mapping, we set a threshold, *m*, and require mapping to satisfy *M*_*ij *_≥ *m*.

Next consider mapping the Pfam domain *f*_*j *_to all possible SCOP domains. We look at the *j*-th column of *M*. The number of nonzeros, , in the column indicates how many SCOP domains could be mapped to. The most likely SCOP domain  that *f*_*j *_will map to is



The threshold *m *is again used to reduce insignificant mapping.

## Results

### Domain mapping

A total of 2081 Pfam families and 2512 SCOP domain families are defined in the set of 8259 PDB protein chains. The average lengthes of Pfam families and SCOP domains are 96 and 174 residues, respectively. The threshold *m *for mapping scores is empirically set to be 0.01 to include as much mapping as possible here, because even a small portion of the overlapping may be informative.

**Figure 3:**
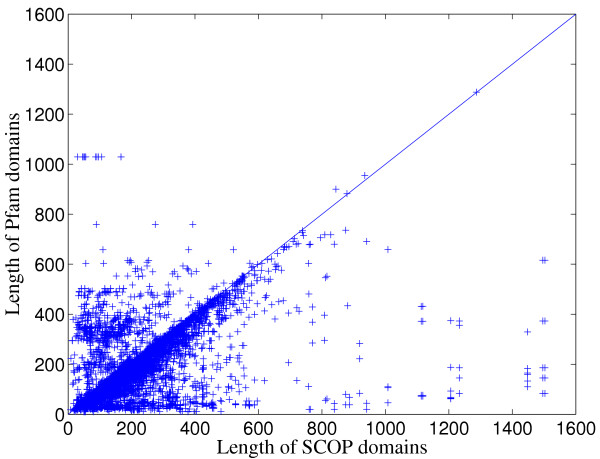
The lengths of SCOP domains are plotted against the lengths of their corresponding Pfam families based on the mapping. Each mapping is represented by a '+', whose x-axis and y-axis values represent the lengthes of the corresponding SCOP domains and Pfam domains, respectively.

From the mapping results, 2008 (80%) SCOP domain families overlap with at least one Pfam family, and these SCOP domain families correspond to 2075 (99.7%) of the Pfam families. On average, each SCOP domain maps to 1.3 Pfam families, and each Pfam domain maps to 1.0 SCOP families. This result is expected because Pfam domains are overall 16% shorter than SCOP domains. The lengths of protein domains in SCOP are plotted against those of the corresponding Pfam families in Figure 3. One-fifth (504) of SCOP domain families have no Pfam counterpart, while only six (0.03%) Pfam families are not mapped to SCOP domain families (Table 1). Further analysis reveals that all the sequence segments corresponding to the unmapped Pfam families represent regions of residues that were absent in the PDB structures. That is, all Pfam families with known PDB structures are mapped to at least one SCOP domain family. It is unclear why 20% of SCOP domain families do not correspond to any Pfam family. One possible explanation is that the there are too few examples of those SCOP domain families to build HMM-profiles for Pfam families.

### Exploring the mapping results

Several types of sequence-structure domain relationships emerge during this study, including:

• One SCOP domain family maps to exactly one Pfam family, where the SCOP domain family and the Pfam family overlap with and only with each other. However, their member sequences and their coverages at each individual sequence may slightly differ.

• One SCOP domain family maps to many Pfam families, where for each member sequence, the coverage of the SCOP domain family corresponds to the summation of those corresponding Pfam families.

• Many SCOP domain families map to one Pfam family, where for each member sequence, the coverage of the Pfam family corresponds to the summation of those corresponding SCOP domain families.

• One SCOP domain family maps to sets of Pfam families, where the SCOP domain family corresponds to one Pfam family at each member sequence, but to different Pfam families at different member sequences.

• Sets of SCOP domain families map to one Pfam family, where the Pfam family corresponds to one SCOP domain family at each member sequence, but to different SCOP domain families at different member sequences.

Examples of each type are provided in Table 2. We present below a detailed analysis of our findings.

#### One-to-one exact mapping

996 SCOP domains each maps to exactly one Pfam family. That is, 39.65% of SCOP domain families and 47.86% of Pfam families have exactly one counterpart in the other type of domain classification. Among these Pfam families, 431 (43.3%) are labelled as 'Family' type, 558 (56.0%) are associated with 'Domain' type, 4 (0.4%) with 'Repeat' type and 3 (0.3%) with 'Motif' type. Thus, the SCOP domain families largely (99.3%) correspond to 'Family' or 'Domain' types in Pfam.

**Figure 4:**
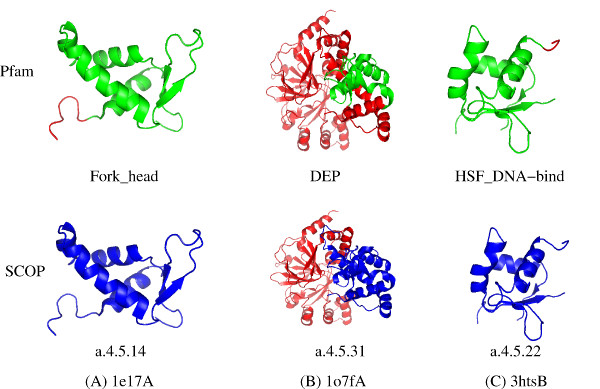
Examples of one-to-one exact mapping between Pfam families and SCOP domain families. The domains are graphed onto the PDB structures of their corresponding member proteins using Pymol. The first row shows Pfam domains and the second row shows their corresponding SCOP domains. The structure regions of Pfam domains are marked in green and those of SCOP domains are marked in blue. Red regions lie outside the SCOP or Pfam domains. The differences in the domain coverage on the structures indicate disagreement between the domain definitions. The differences are usually in domain boundaries. The PDB proteins 1e17A, 1o7fA, and 3htsB are used for the illustration.

In the case of one-to-one mapping, these Pfam domains have an average length of 164.0, and the SCOP domains have an average length of 182.7, 11% longer on average than the corresponding Pfam domains. Even where two domains are mapped one-to-one, their definitions may slightly disagree. For instance, their member protein sequences may not be exactly the same, or their corresponding sequence segments may not completely overlap. A few examples of Pfam domains and SCOP domains are graphed onto the corresponding member protein structures using Pymol [20] as shown in Figure 4 to illustrate the latter case.

**Figure 5:**
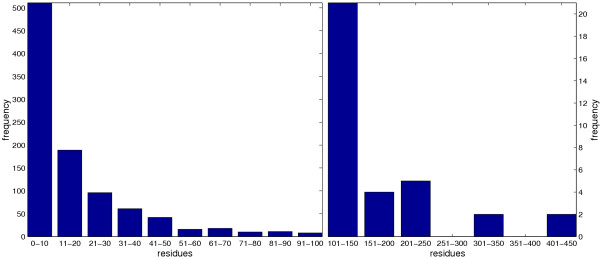
Histogram of differences in the endpoints of the domains. The differences in the endpoints show a power law distribution: more than 50% of the mappings between Pfam families and SCOP domain families differ by less than 10 residues and only 3.4% mapped domains differ by more than 100 residues.

**Figure 6:**
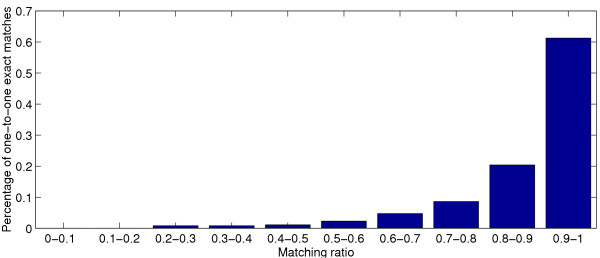
Distribution of the mapping ratio for one-to-one exact mapping. The mapping ratios are calculated with Eq. 2. Among the cases of one-to-one exact mapping, 61.24% have a mapping ratio larger than 0.9, 81.62% have a mapping ratio larger than 0.8, and 90.26% have a mapping ratio larger than 0.7.

Figure 5 shows the histogram of the differences in domains' endpoints. For two domains *f*_*i *_and *s*_*j*_, their difference in the endpoints is calculated as the total length of the regions covered by *f*_*i *_or *s*_*j *_minus the length of the shared regions covered by *f*_*i *_and *s*_*j*_. More than 50% (511) of the mappings between Pfam families and SCOP domain families differ by less than 10 residues, while only 3.4% (34) of domain mappings differ by more than 100 residues. To quantify the extent of the one-to-one mapping, we define a mapping ratio as



where *P *is the common member protein sequences of the two types of domain families, *intersect*(, ) is the length of the overlapped portion of the *i*th Pfam family with the *j*th SCOP domain family at the *k*th member protein sequence, and *union*(, ) is the length of the regions covered by either of them. Figure 6 shows the distribution of the mapping ratios. Among these cases of one-to-one mapping, 61.24% have a mapping ratio larger than 0.9. That is, the two types of domain definitions vary in less than 10% of the domain sequences. 81.62% vary in less than 20% of the domain sequences, and 90.26% vary in less than 30% of the domain sequences.

#### One SCOP domain family to many Pfam families

**Figure 7:**
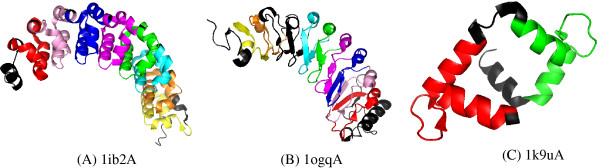
Structures of SCOP domains each mapped to several copies (repeats) of a Pfam family. The corresponding Pfam families may be of type 'Family', 'Domain', or 'Repeat'. PDB proteins lib2A, 1ogqA, and lk9uA are used for the illustration. (A) SCOP domain *a*.118.1.8 (*Pumilio repeat*) corresponds to 8 copies of Pfam family *PUF *(*Pumilio-family RNA binding repeat*) of type 'Family'. The regions marked by red, pink, blue, purple, green, cyan, orange, and yellow each represent a copy of *PUF*. (B) SCOP domain *c*.10.2.8 (*Polygalacturonase inhibiting protein PGIP*) corresponds to 8 copies of Pfam family *LRR *(*Leucine Rich Repeat*) of type 'Repeat'. The eight copies of *LRR *are each marked with a unique color: red, pink, blue, purple, green, cyan, orange, and yellow. (C) SCOP domain *a*.39.1.10 (*Polcalcin phl p 7*) corresponds to 2 copies of Pfam family *efhand *(*EF hand*) of type 'Domain'. The two copies of *efhand *are marked in red and green, respectively.

A total of 76 SCOP domain families map to multiple Pfam families. About half (33) of these SCOP domain families correspond to several copies (repeats) of the same Pfam family. The corresponding Pfam families may be of Pfam type 'Family', 'Domain', or 'Repeat'. One example is provided for each case in Figure 7. SCOP domain *a*.118.1.8 (*Pumilio repeat*)corresponds to 8 copies of Pfam family *PUF *(*Pumilio-family RNA binding repeat*) of type 'Family' (Figure 7(A)), SCOP domain *c*.10.2.8 (*Polygalacturonase inhibiting protein PGIP*) corresponds to 8 copies of Pfam family *LRR *(*Leucine Rich Repeat*) of type 'Repeat' (Figure 7(B)), and SCOP domain *a*.39.1.10 (*Polcalcin phl p *7) corresponds to 2 copies of Pfam family *efhand *(*EF hand*) of type 'Domain' (Figure 7(C)). It seems that these Pfam families all serve as building blocks for SCOP domains and more careful investigation is required to determine the validity of these domains.

Several Pfam families, such as *LRR *(*Leucine Rich Repeat*) and *efhand *(*EF hand*) have a high frequency of mapping to SCOP domain families. For instance, the SCOP domain *c*.10.1.2(*Rna1p (RanGAP1), N-terminal domain*) maps to two copies of the Pfam family *LRR*, the SCOP domain *c*.11.1.1 (*Outer arm dynein light chain 1*) maps to four copies of *LRR*, and the SCOP domain *c*.10.2.8 (*Polygalacturonase inhibiting protein PGIP*) maps to eight copies of *LRR *(Figure 7(B)). Most of the SCOP counterparts of *LRR *belong to the SCOP *L domain-like *superfamily. Pfam annotates *LRR *as *Repeat *type, and describes them as 'short sequence motifs present in a number of proteins with diverse functions'. These types of Pfam families actually represent structural components that form structural domains. They differ from domains in that they are functionally and evolutionarily dependent on other structure components. Therefore, we would suggest these Pfam families being removed from the Pfam-A family.

#### Many SCOP domain families to one Pfam family

**Figure 8:**
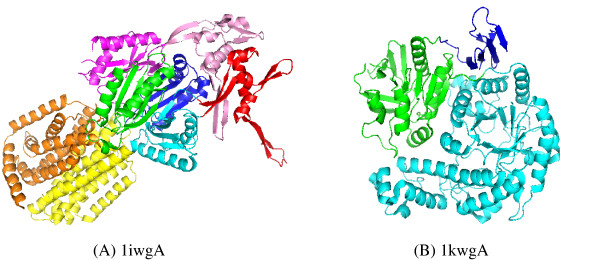
A series of SCOP domains are mapped to a Pfam family. (A) The Pfam family *ACR_tran *(*AcrB/AcrD/AcrF family*) corresponds to eight SCOP domain families for PDBID 1iwgA, three of which are unique. The regions marked with red and pink are two copies of the SCOP domain family *d*.225.1.1 (*Multidrug efflux transporter AcrB TolC docking domain; DN and DC subdomains*), marked with yellow and orange are two copies of the SCOP domain family *f*.35.1.1 (*Multidrug efflux transporter AcrB transmembrane domain*), and the rest are four copies of the SCOP domain family *d*.58.44.1 (*Multidrug efflux transporter AcrB pore domain; PN1, PN2, PC1 and PC2 subdomains*). (B) The Pfam family *Glyco_hydro_42 *(*Beta-galactosidase*) mapped to a series of the SCOP domain families {*c*.1.8.1 (*Amylase*, *catalytic domain*), *c*.23.16.5 (*A4 beta-galactosidase middle domain*), *b*.71.1.1 (*alpha-Amylases*, *C-terminal beta-sheet domain*)} in PDB protein 1kwgA. They are marked in cyan, green and blue, respectively.

There are 106 Pfam families mapped to multiple SCOP domains. Of them, 25 map to repeats of the same SCOP domain. Several examples for this type of mapping are shown in Figure 8. According to the mapping results for the bacterial multidrug efflux transporter AcrB (PDB ID 1iwgA), the Pfam *ACR_tran *(*AcrB/AcrD/AcrF*) family corresponds to eight SCOP domain families in the order of *f*.35.1.1 (*Multidrug efflux transporter AcrB transmembrane domain*), *d*.58.44.1 (*Multidrug efflux transporter AcrB pore domain; PN1, PN2, PCI and PC2 subdomains*), *d*.58.44.1, *d*.225.1.1 (*Multidrug efflux transporter AcrB TolC docking domain; DN and DC subdomains*), *f*.35.1.1, *d*.58.44.1, *d*.58.44.1, and *d*.225.1.1 (Figure 8(A)). Among these SCOP domains, only three are unique, and the second four SCOP domains are exact repeats of the first four SCOP domains. These SCOP domains are found to co-exist in PDB protein chains 1iwG, 1oy8, 1oyE, 1oy6, 1oy9, and 1oyD based on SCOP records. Further inspection reveals that these domains are always present together in the multidrug efflux transporter proteins in the same order, and they act collaboratively in the process of exporting toxic compounds out of the cell [21].

However, each functions independently: *d*.225.1.1 docks TolC into AcrB, *f*.35.1.1 translocates substrates from the cell interior, and *d*.58.44.1 translocates substrates into the TolC tunnel. In this sense, the SCOP domain classification is more accurate and the Pfam *ACR_tran *family may be chopped into eight small domains. Similarly, the Pfam family *Glyco_hydro_42 *(*Beta-galactosidase*), mapped to a series of the SCOP domain families *c*.1.8.1 (*Amylase, catalytic domain*), *c*.23.16.5 (*A4 beta-galactosidase middle domain*), and *b*.71.1.1 (*alpha-Amylases*, *C-terminal beta-sheet domain*), may be partitioned into three small domains.

#### One SCOP domain to sets of Pfam families

**Figure 9:**
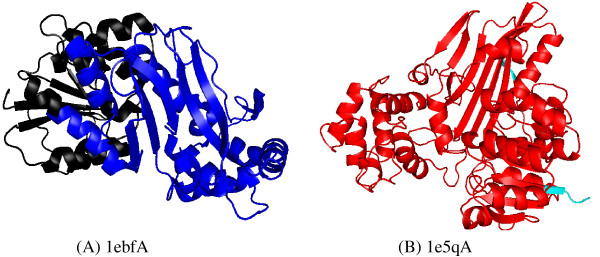
One SCOP domain mapped to different sets of Pfam families. (A) The SCOP domain *d*.81.1.2 is mapped to the Pfam family *Homoserine-dh *(marked in blue) in PDB protein lebfA. (B) The SCOP domain *d*.81.1.2 is mapped to the Pfam family *Saccharop-dh *(marked in red) in PDB protein le5qA.

289 SCOP domains are mapped to sets of Pfam domains, one set at a time. For example, the SCOP domain *d*.81.1.2 (*Homoserine dehydrogenase-like*) maps to the Pfam family *Homoserine_dh *(*Homoserine dehydrogenase*) on the PDB protein chain 1*ebf A *(Figure 9(A)) and to the Pfam family *Saccharop_dh *(*Saccharopine dehydrogenase*) on the PDB protein chain 1*e5qA *(Figure 9(B)). Another example is the SCOP domain family *e*.8.1.1 (*DNA polymerase I*) which maps to the Pfam *DNA_pol_A *(*DNA polymerase family A*) and *DNA_pol_B *(*DNA polymerase family B*) on different PDB protein chains. Relationships are suggested between these Pfam families that are individually mapped to a same SCOP domain family. If several sets of Pfam families are mapped to the same SCOP domain, based on the fact that the SCOP domain families are functionally independent, these Pfam families are very likely to share both functions and structures. Therefore, close scrutiny may be required to determine whether these Pfam families should be merged or not.

#### Sets of SCOP domain families to one Pfam family

We find 314 Pfam families that map to multiple sets of SCOP domain families. Under this category a subtype of special interest is Pfam families corresponding to SCOP superfamilies. Some examples of this subtype are listed in Table 3. For instance, the SCOP domain families *c*.107.1.1 (*Manganese-dependent inorganic pyrophosphatase *(*family II*)) and *c*.107.1.2 (*Exonuclease RecJ family*) each individually map to the Pfam family *DHH *(*DEE Family*). Both of the SCOP domains belong to the SCOP superfamily *c*.107.1 (*DHH phosphoesterases*). Another example is the Pfam family *Glyoxalase *(*Glyoxalase/Bleomycin resistance protein/Dioxygenase superfamily*). The Pfam domain is independently mapped to the following four SCOP domain families: *d*.32.1.1 (*Glyoxalase I *(*lactoylglutathione lyase*)), *d*.32.1.2 (*Antibiotic resistance proteins*), *d*.32.1.3 (*Extradiol dioxygenases*), and *d*.32.1.4 (*Methylmalonyl-CoA epimerase*). These SCOP domains all belong to the SCOP superfamily *d*.32.1 (*Glyoxalase/Bleomycin resistance protein/Dihydroxybiphenyl dioxygenase*). From the Pfam annotation of the Pfam family *Glyoxalase*, we see that Pfam seems to be aware of it is a superfamily. But the flat organization of Pfam fails to reflect this property explicitly. In this sense, the comparative mapping between SCOP and Pfam could help Pfam to build a hierarchical organization. On the other hand, it is known that all SCOP classes higher than 7 are considered "not true SCOP classes" and their subtypes (folds, superfamilies, and families) are considered not "true", either. We can utilize this type of mapping to put those SCOP domains in meaningful classes. For example, the SCOP domain families *c*.96.1.1 (*Fe-only hydrogenase*) and *i*.4.1.1 (*Electron transport chains*) each individually map to the Pfam family *Fe_hyd_lg_C *(*Iron only hydrogenase large subunit, C-terminal domain*). It may be inferred that the SCOP domain family *i*.4.1.1 is related to SCOP superfamily *c*.96.1.

#### Combination of types

**Figure 10:**
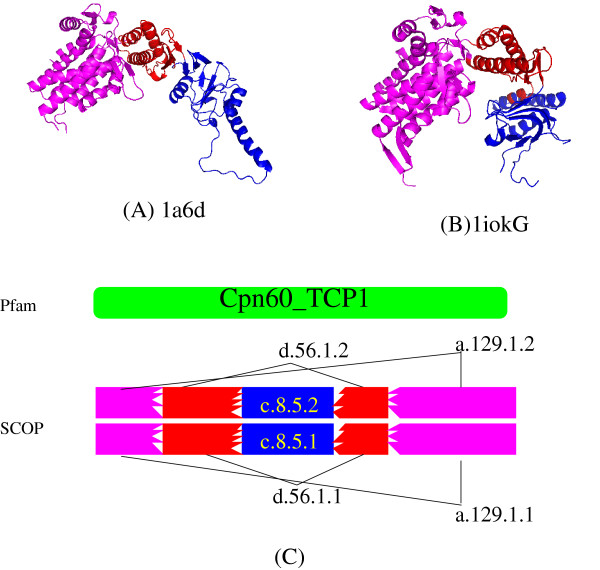
A Pfam family corresponds to two different sets of SCOP domains, each consisting of a series of three domains. The PDB proteins 1a6d and 1iokG are used for the illustration. The SCOP domains a.129.1.1 and a.129.1.2 are marked in purple. The SCOP domains d.56.1.1 and d.56.1.2 are marked in red. The SCOP domains c.8.5.1 and c.8.5.2 are marked in blue. The Pfam domain *Cpn60_TCP*1 is marked in green. (A)The Pfam family *Cpn60_TCP1 *is mapped to the set of SCOP domain families: {a.129.1.2 + d.56.1.2 + c.8.5.2}. (B)The Pfam family *Cpn60_TCP1 *is mapped to the set of SCOP domain families: {a.129.1.1 + d.56.1.1 + c.8.5.1} (C)Illustration of the insertion process which supports the SCOP domain definitions for this particular case. The SCOP domain families *a*.129.1.1 and *a*.129.1.2 are the parent domains. Later the SCOP domain families *d*.56.1.1 and *d*.56.1.2 are inserted into *a*.129.1.1 and *a*.129.1.2, respectively. Finally the SCOP domain *c*.8.5.1 is inserted into *d*.56.1.1, and the SCOP domain *c*.8.5.2 is inserted into *d*.56.1.2.

In many cases, a combination of several types is observed. For example, the Pfam *TCP-1/cpn60 chaperonin *(*Cpn60.TCP1*) family is mapped to two different sets of SCOP domains, each consisting of a series of three domains: {*a.129.1.2 (Group II chaperonin (CCT, TRIC), ATPase domain), d.56.1.2 (Group II chaperonin (CCT, TRIC), intermediate domain), and c.8.5.2 (Group II chaperonin (CCT, TRIC), apical domain*)} and {*a.129.1.1 (GroEL chaperone, ATPase domain), d.56.1.1 (GroEL-like chaperone, intermediate domain), and c.8.5.1 (GroEL-like chaperone, apical domain*)}. These two sets of SCOP domains usually occur together. However, the SCOP domain families *c*.8.5.1 and *c*.8.5.2 are also each present on their own in many PDB protein chains. This indicates that *c*.8.5.1 and *c*.8.5.2 are each an independent, single domain. According to Aroul-Selvam *et. al *[22], this three domain set is formed through two insertions as follows: *a*.129.1.1 and *a*.129.1.2 are the parent domains, followed by the insertion of *d*.56.1.1 into *a*.129.1.1 and *d*.56.1.2 into *a*.129.1.2. Finally*c*.8.5.1 is inserted into *d*.56.1.1, and *c*.8.5.2 is inserted into *d*.56.1.2 (Figure 10). Members with the domain organization of {*a.129.1.2*, *d.56.1.2*, *c.8.5.2*} are the molecular chaperone GroEL and proteins with similar functions. These proteins are known to have three functional domains: equatorial (ATPase) domain, intermediate domain, and apical domain, each with its own distinct function. The whole protein functions as a molecular chaperone, which binds unfolded polypeptides *in vitro*, and has a weak ATPase activity. The apical domain is involved in substrate binding. The equatorial domain contains the nucleotide binding site and provides most of the intersubunit contacts. The linker domain serves to transmit allosteric effects between the other two domains.

### Comparative mapping may help build Pfam clans

The Pfam database employs a flat organization, with a 'Type' annotation attached to each family. The annotation is to some extent similar to levels in SCOP hierarchical organization. Clans have been introduced in Pfam to reflect the evolutionary relationship between different families. Each clan contains two or more Pfam families that have arisen from a single evolutionary origin. However, Pfam release 14.0 contains only 15 clans covering less than 100 Pfam families. With our comparative mapping results, the SCOP hierarchy may be used to help Pfam generate the clans. For example, when one SCOP domain family is mapped to sets of Pfam families, a strong connection/relationship between those Pfam domains may be implied. A clan may be inferred from those Pfam families. Therefore, we compared our results with the existing Pfam clans. Table 4 lists the member families in existing Pfam clans and their corresponding SCOP domains. We only list 10 rather than 15 because the other five mostly contain Pfam families not used in the comparison. As can been seen from the Table, members of a clan usually correspond to a SCOP family or a SCOP superfamily. Therefore, we believe the results from comparative mapping could potentially be helpful in building Pfam clans.

### Phylogenetic analysis

Domains are considered evolutionarily independent units, and the evolution history of each domain is expected to be characteristic. Similar domain evolutionary histories may indicate relations among domains. Therefore, we propose to use correlation in domain evolution to validate the domain definitions by Pfam and SCOP in the case of disagreement.

Tan *et. al *have designed a tool to compute the similarities between proteins' evolutionary histories [23]. This approach can be slightly modified to fit our needs for determining the similarities between domains' evolutionary histories. We define the evolutionary correlation between two domains as the average correlation between pairs of their member sequences. The correlation between two sequence segments is then defined as the Pearson correlation coefficient of the evolutionary distance matrices of the two sequences. It is computed using the following steps. First, Blastp is used to find the orthologous protein sequences in two sets of genomes; bacterial and eukaryotic. The bacterial data set contains proteins from the genomes of eighteen species: *Acinetobacter sp ADP1, Fusobacterium nucleatum, Nitrosomonas europaea, Vibrio parahaemolyticus, Bacillus anthracis Ames, Geobacter sulfurreducens, Pyrococcus abyssi, Xylella fastidiosa, Campylobacter jejuni, Helicobacter hepaticus, Rickettsia conorii, Yersinia pestis KIM, Deinococcus radiodurans, Lactococcus lactis, Streptococcus pyogenes, Escherichia coli K12, Methanosarcina mazei, and Thermotoga maritima*. The eucaryotic data set contains genome protein sequences from nine species, including *Arabidopsis thaliana, Encephalitozoon cuniculi, Plasmodium falciparum, Caenorhabditis elegans, Homo sapiens, Rattus norvegicus, Drosophila melanogaster, Mus musculus, and Saccharomyces cerevisiae*.

Second, for each species, the orthologous protein sequence with the highest E-value is selected (if a significant one exists). Third, ClustalW is then used to align these sequences. Fourth, the Pearson correlation coefficient of those mapping matrices is computed with Equation 3, which represents the correlation between the corresponding sequence pair.



where *N *is the number of species where orthologous sequences were retrieved, *S *and *P *are *N *× *N *distance matrices from ClustalW alignment of sequence segments in SCOP domain families and Pfam families, respectively. The correlation between two domains is then expressed as:



where *abs *(*x*) gives the absolute value of *x*, and *N*_*i *_and *N*_*j *_are the number of member sequences for domains *i *and *j*, respectively.

**Figure 11:**
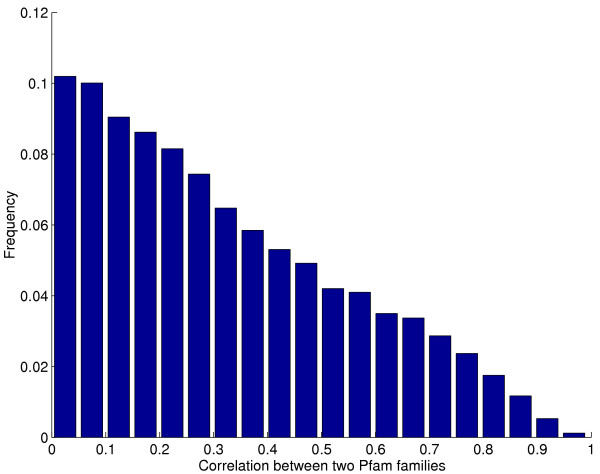
Distribution of correlations between two Pfam domains. The Pfam families are randomly selected and their correlation is calculated as described in Section *Phylogenetic Analysis*. The correlation represents the relatedness of two domains. Its value ranges from 0 to 1, with 1 indicating 100% similarity in the two domains' evolutionary histories and 0 no similarity. Genome protein sequences from bacteria are used in the computation. About 76% of the domain pairs have a correlation less than 0.5.

This correlation measures the relatedness of the two domains. Its value ranges from 0 to 1, where 1 means 100% similarity in the two domains' evolutionary histories and 0 means no similarity. Now we need to determine the lower threshold of the correlation which indicates co-evolution. We randomly select two Pfam families and compute their correlation. Similarly, the random correlation between two SCOP domains is calculated. The distributions of the correlations are shown in Figure 11.

When multiple Pfam families are mapped to a SCOP domain, we compute the evolutionary correlation of these Pfam families. The correlation may suggest whether those Pfam families should be merged or not. If two domains reside on the same set of sequences in close vicinity and share the same set of evolutionary characteristics, then we propose those domains should be considered as co-evolved and treated as a single, larger domain. Thus, domain definitions may depend on the relative evolutionary histories.

## Conclusion

In this paper, we discuss the comparative mapping of structure-based domains to sequence-based domains in order to address the question of how each of these models individually captures the evolutionary, structural and functional features of protein domains. The ultimate purpose of our comparative mapping is to provide insight into protein domain definitions.

Using domain definitions from SCOP and Pfam, we mapped the two types of domain definitions to each other using their location information for each domain instance. Mapping results reveal a general agreement between the two types of domain definitions. To further analyze the problem, we introduce several subcategories (one/many SCOP domain to one/many Pfam domain, and vice versa), and provide detailed studies of the mapping using examples from each category.

In the subcategory of one SCOP to/from one Pfam mapping, often the mapping is not perfect: the two domains only partially overlap. Analysis shows that around 62% of the cases of one-to-one mapping agree on 90% or more of their coverage. The differences are usually in the domain boundaries. This result suggests that evolutionary history of the mapped region versus the unmapped region may be examined to see how those unmapped portions are evolutionarily related to the mapped region.

In many cases, a SCOP domain family is mapped to a series of repeats of a Pfam family. These Pfam families, such as *LRR*, are more likely domain components without the properties of structural domains. Therefore, we would suggest Pfam remove those families. The mapping results could also be used to infer classification for SCOP domain families that do not belong to the true classes (classes larger than 7). For example, in the cases that a set of SCOP domains are mapped to one Pfam family, structural and functional relationships are suggested among the set of SCOP domains. This information may be useful for the assignment of SCOP domains to true SCOP classes. On the other hand, the Pfam database employs a flat organization and fails to indicate the relationship between Pfam families. Although Pfam introduced clans to reflect the relationship between different families, the building of clans needs input from experts and as a result, there only 15 clans in Pfam release 14.0. Our comparison of the mapping results with the Pfam clans showed that members of a clan usually correspond to a SCOP family or a SCOP superfamily.

Therefore, the comparative mapping results may be used to help Pfam generate the clans. Perhaps most interesting, several sharp disagreements between SCOP domain families and Pfam families have been discovered, and studied in some detail. Further examination of those domain families using phylogenetic analysis would be beneficial. We have proposed using evolutionary correlation between domains to measure the fitness of the domain classification. Clearly, further studies on these sharp differences are necessary and future research may be targeted in this area.

## Authors' contributions

SRH conceived and coordinated the study. YZ participated in the design of the study, implemented the comparative mapping methods, performed the data and statistical analyses, as well as drafted the manuscript. SRH, JMC, and CD participated in experimental design, and edited the final draft of the manuscript. All authors read and approved the manuscript.

## Figures and Tables

**Table 1:** Pfam families with no corresponding SCOP domain families. The annotations for Pfam families were retrieved from the Pfam database.

Pfam family	Type	Annotation
Cytochrom_B559a	Family	The lumenal portion of cytochrome b559 alpha chain.
MHC_I_C	Family	The C-terminal region of the MHC class I antigen.
STN	Family	Found at the N-terminus of the Secretins of the bacterial type II/III secretory system as well as the TonB-dependent recep- tor proteins, which are involved in TonB-dependent active up- take of selective substrates.
Phe_tRNA- synt_N	Domain	Aminoacyl tRNA synthetase class II, N-terminal domain.
RNA_pol_Rpb1_R	Repeat	The repetitive C-terminal domain (CTD) of Rpb1 (RNA poly-merase Pol II).
Prion_octapep	Repeat	Found at the amino terminus of prion proteins and shown to bind to copper.

**Table 2:** Types of mapping between SCOP and Pfam families.

Type of map	Example	
	
	SCOP	Pfam
One SCOP domain family to exactly one Pfam family	b.81.2.1	CfAFP
One SCOP domain family to a series of Pfam families	e.38.1.1	{PCRF, RF-1}
A series of SCOP domain families to one Pfam family	{d.179.1.1, d.58.20.1}	HMG-CoA_red
A SCOP domain family to several sets of Pfam families	b.41.1.1	{PRCH, PRC}; PRC
Sets of SCOP domain families to one Pfam family	{f.10.1.1, b.1.18.4}; i.6.1.1	Alpha_E1_glycop

**Table 3:** Examples for cases where a Pfam family corresponds to a SCOP superfamily.

Pfam	Type	SCOP
DHH	Family	c.107.1.1; c.107.1.2
OsmC	Family	d.227.1.2; d.227.1.1
Pec_lyase_C	Domain	b.80.1.2; b.80.1.1
Glyoxalase	Domain	d.32.1.3; d.32.1.1; d.32.1.4; d.32.1.2
TOBE	Domain	b.40.6.1; b.40.6.3; b.40.6.2
HhH-GPD	Domain	a.96.1.2; a.96.1.3; a.96.1.1
NAD_binding_1	Domain	c.25.1.4; c.25.1.1; c.25.1.5; c.25.1.2
Glyco_hydro_15	Family	a.102.1.1; a.102.1.5
Ricin_B_lectin	Repeat	b.42.2.1; b.42.2.2
Prenyltrans	Repeat	a.102.4.3; a.102.4.2
HHH	Motif	a.60.2.1; a.60.4.1; a.60.2.3; a.60.2.2

**Table 4:** Members of Pfam clans and their corresponding SCOP domains.

Clan ID	Member families	Corresponding SCOP domains
1	Laminin_EGF	g.3.11.2
	EGF_CA	g.3.11.1
	EGF	g.3.11.1
2	Laminin_G_2	b.29.1.4
	Laminin_G_1	b.29.1.4
3	Kazal_2	g.15.1.1
	Kazal_1	g.15.1.1
4	KH_1	d.52.3.1
	KH_2	d.52.3.1
5	SNF2_N	-
	ResIII	c.37.1.19
	Flavi_DEAD	-
	DEAD_2	-
	DEAD	c.37.1.19
6	ENTH	a.118.9.1
	ANTH	a.118.10.1
7	SH3_2	b.34.2.1
	SH3_1	b.34.2.1
8	V-set	b.1.1.1
	Ig	b.1.1.1
	I-set	b.1.1.1
	C2-set	b.1.1.2
	C1-set	b.1.1.3
9	TAFII28	a.22.1.3
	TAF	a.22.1.3
	Histone	a.22.1.3
	CBFD_NFYB_HMF	a.22.1.3
10	Transpeptidase	e.3.1.1
	Peptidase_S11	e.3.1.1
	Lactamase_B	-
	Betalactamase	e.3.1.1
